# In vitro progesterone modulation on bacterial endotoxin-induced production of IL-1β, TNFα, IL-6, IL-8, IL-10, MIP-1α, and MMP-9 in pre-labor human term placenta

**DOI:** 10.1186/s12958-015-0111-3

**Published:** 2015-10-07

**Authors:** G. Garcia-Ruíz, P. Flores-Espinosa, E. Preciado-Martínez, L. Bermejo-Martínez, A. Espejel-Nuñez, G. Estrada-Gutierrez, R. Maida-Claros, A. Flores-Pliego, Veronica Zaga-Clavellina

**Affiliations:** Inmunobiochemistry Branch, Instituto Nacional de Perinatología “Isidro Espinosa de los Reyes”, Montes Urales 800, Lomas Virrreyes, Ciudad de Mexico, 11000 Mexico; Neonatology Branch, Instituto Nacional de Perinatología “Isidro Espinosa de los Reyes”, Montes Urales 800, Lomas Virreyes, Ciudad de Mexico, 11000 México; Facultad de Estudios Superiores Cuautitlán, Universidad Nacional Autónoma de México, Estado de Mexico Ciudad de Mexico, 54700 Mexico

**Keywords:** Progesterone, Cytokines, Metalloproteinase, Bacterial endotoxin, Inflammation, Human placenta, Intrauterine infection, Preterm labor

## Abstract

**Background:**

During human pregnancy, infection/inflammation represents an important factor that increases the risk of developing preterm labor. The purpose of this study was to determine if pre-treatment with progesterone has an immunomodulatory effect on human placenta production of endotoxin-induced inflammation and degradation of extracellular matrix markers.

**Methods:**

Placentas were obtained under sterile conditions from pregnancies delivered at term before the onset of labor by cesarean section. Explants from central cotyledons of 10 human placentas were pre-treated with different concentrations of progesterone (0.01, 01, 1.0 μM) and then stimulated with 1000 ng/mL of LPS of *Escherichia coli*. Cytokines TNFα, IL-1β, IL-6, IL-8, MIP-1α, IL-10 concentrations in the culture medium were then measured by specific ELISA. Secretion profile of MMP-9 was evaluated by ELISA and zymogram. Statistical differences were determined by one-way ANOVA followed by the appropriate ad hoc test; *P* < 0.05 was considered statistically significant.

**Results:**

In comparison to the explants incubated with vehicle, the LPS treatment led to a significant increase in the level of all cytokines. In comparison to the explants treated only with LPS, pre-treatment with 0.01–1.0 μM progesterone significantly blunted (73, 56, 56, 75, 25, 48 %) the secretion of TNF-α, IL-1β, IL-6, IL-8, MIP-1α, IL-10, respectively. The MMP-9 induced by LPS treatment was inhibited only with the highest concentration of progesterone. Mifepristone (RU486) blocked the immunosuppressive effect of progesterone.

**Conclusions:**

The present results support the concept that progesterone could be part of the compensatory mechanism that limits the inflammation-induced cytotoxic effects associated with an infection process during gestation.

## Background

The cervicovaginal/intrauterine infection process during pregnancy represents a condition of extreme vulnerability for the mother and fetus. The immunologic defense process induces a pro-inflammatory environment that jeopardizes/disrupts the immune privilege of the intrauterine cavity.

There is evidence that almost 30 % of women with preterm labor have microbial invasion or inflammation of the amniotic cavity [1, 2]; this condition induces uncontrolled production and increase of Th1 cytokines such as interleukin (IL)-1β, tumor necrosis factor (TNF)α, and IL-6 that alter the intra-amniotic milieu, leading to disruption of fetal tolerance [3].

Evidence supports the existence of a strong relation between the microorganisms that reach the amniotic cavity from the vagina and bacteria identified in the fetal circulation of premature neonates [4]. In this adverse scenario, the placenta represents a physical barrier that protects the product.

The placenta allows for the diffusion of nutrients and oxygen from the maternal blood to the fetal blood; therefore, this tissue is key in the immune-endocrine relationship between mother and fetus, creating an immune tolerance that permits their co-existence along 40 weeks.

From the 7th week of gestation, the placenta takes over steroid production and becomes the main source of steroid hormone until the end of pregnancy [5]. Progesterone (P4) is a steroid hormone that modulates/regulates different biological processes in a broad range of tissues, its action is essential in different reproductive events, such as ovulation, uterine and mammary gland development. Its function is essential during the establishment and maintenance of pregnancy and the onset of labor.

Both clinical and experimental data support the concept that normal pregnancy is a Th-2-like phenomenon. It is now evident that the protection of the fetus against a harmful maternal immune response is based on a complicated mechanism, and the communication between the various steps in the cascade of events is accomplished via cytokines.

Cytokines have been shown to affect the outcome of pregnancy, several pro-inflammatory cytokines, including TNF-α, IL-1β, IL-6, have been implicated in the onset of spontaneous preterm labor [6–9]. The biological significance of this immunologic response includes deep alterations in the maternal immune system, as well as the establishment of a fetal inflammatory response syndrome that has been described in preterm birth and is strongly associated with an adverse perinatal outcome [10–12].

The toxic effects of inflammation lie in the well-known fact that a strong cellular anti-fetal response is deleterious for pregnancy. Under these pathological conditions, the maternal-fetal unit displays compensatory mechanisms that limit partially the effects of pro-inflammation and privilege the continuity of gestation.

Among its multiple functions, P4 elicits immune-modulatory effects creating a suitable immune environment. Although the mechanism of action has not been completely characterized, experimental and clinical evidence indicates that P4 elicits anti-inflammatory properties. Likewise, there is evidence to support that prevention of the pro-inflammatory process by this hormone may be exerted through modulation of the host immune response [13–15].

The present work was conducted to determine whether P4 could modulate the secretion of TNFα, IL-1β, IL-6, IL-8, IL-10, and matrix metalloproteinase (MMP)-9 in explants of human placentas.

## Methods

### Reagents

Progesterone (4-pregnene-3, 20-dione), LPS (from *Escherichia coli* 055:B5), and RU486 (mifepristone) were purchased from Sigma (St Louis, MO, USA).

### Biological samples

The present project was approved by both the Human Ethical and Research Committees of the *Instituto Nacional de Perinatologia “Isidro Espinosa de los Reyes”* (INPer IER- 212250-06161) in Mexico City.

Ten placentas were collected from healthy women, 21–35 years, with normal, uncomplicated, singleton pregnancies, who underwent elective cesarean section at term (37–39 weeks of pregnancy) with no evidence of active labor, cervical dilatation or loss of mucus plug.

Written informed consents were obtained from all participants, their care was provided at the obstetrics outpatient service of the INPer IER. Patients with antecedents of cervicovaginal infection, chronic hypertension, diabetes mellitus, cardiac or renal insufficiency, or other systemic illnesses were no included in this study.

Immediately after delivery, microbial analyses were conducted to preclude the presence of chorioamniotic infection. Sterile swabs were randomly rolled across selected areas of the placenta. The swabs were then rolled onto Columbia agar with 5 % sheep blood, used as a primary isolation medium for fastidious and non-fastidious aerobic microorganisms. Appropriate selective media were used to detect specific pathogens and only infection-free membranes were used for this study.

Explants of the placenta were transported to the laboratory in sterile Dulbecco’s Modified Eagle Medium (DMEM; Gibco, Life Technologies, CA, USA) supplemented with 100 U/mL penicillin and 100 μg/mL streptomycin (Gibco). Tissues were manipulated under sterile conditions. Two central cotyledons were dissected, once the decidua of the chorion laeve had been removed, 3 explants of 1 cm^3^ were cultured in each well of a 12-well tissue culture plate with 2 mL of DMEM (GIBCO) without phenol red and supplemented with heat-inactivated and hormone-free 10 % fetal calf serum. Then, 1 mM sodium pyruvate and 1X antibiotic-antimycotic solution (100 U/mL penicillin, 100 μg/mL streptomycin, and 2.5 μg/mL amphotericin) were added to each well. The explants were incubated under 5 % CO_2_/ 95 % air at 37 °C.

### Validation of placenta explants culture

To warrant that the explants were metabolically active, their viability was determined by a colorimetric assay using tetrazolium salts added to the culture medium (Boehringer Mannheim, Germany). The assay was performed every 24 h of culture over 4 days (data not shown). To explore the secretion profile of different analytes, a time-response curve was also performed (data not shown)

### Treatment of placenta explants

The first 24 h of culture, the explants were incubated in absence (basal control plus vehicle [0.01 % ethanol]) and presence of three different concentrations (1.0 μM, 0.1 μM, and 0.01 μM) of P4 for 24 h; after this time, fresh medium was added including co-stimulations with 1000 ng/ml of LPS plus 0.01, 0.1, and 1 μM of P4. Another set of experiments was included, co-incubating the explants with LPS plus the highest concentration of P4 and RU-486 (100 μM), controls with LPS, P4, or RU-486 were also included.

### Cytokines quantitation by ELISA

The concentrations of TNFα, IL-1β, IL-6, IL-8, MIP1α, IL-10, and MMP-9 (R&D Systems, Minneapolis, MN, USA) present in cell culture supernatants were determined by sandwich ELISA, using human specific duo-set kits according to manufacturer’s instructions.

To coat the plates, the following capture anti-human antibodies (hAbs) were used: anti-human hAb TNFα (4 μg/mL), anti-human hAb IL-1β (4 μg/mL), anti-human hAb IL-6 (2 μg/mL), anti-human hAb IL-8 (0.5 μg/mL), anti-human hAb MIP-1α (0.4 μg/mL), anti-human hAb IL-10 (2 μg/mL), anti MMP-9 (1 μg/mL).

For the TNF-α assay, a standard curve was developed from 0.5 to 10 ng/mL with a sensitivity of 0.2 ng/mL; for the IL-1β assay, from 3.00 to 250 pg/mL; for the IL-6 assay, the curve was linear from 0.5 to 10 ng/mL with a sensitivity of 0.2 ng/mL; for IL-8, the curve was developed from 15.6 to 1000 pg/mL with sensitivity of 10 pg/mL; for MIP-1α, the curve was developed from 7.4 to 1000 pg/mL; and for IL-10, from 31.25 to 2000 pg/mL with a sensitivity of 10 pg/mL. The MMP-9 curve was performed from 31.2 to 2500 pg/mL.

### Zymography

SDS-polyacrylamide gels (9 %) co-polymerized with porcine gelatin (1 mg/mL) were prepared according to the standard methods previously described by [16]. Briefly, 5 μg of each supernatant and tissue lysate sample were loaded into each well under non-denaturing conditions and run under a constant current (10 mA) for 1.6 h; then, gels were washed in 2.5 % Triton X-100 for 0.5 h and incubated overnight at 37 °C in an activation buffer (50 mM Tris pH 7.4, 0.15 M NaCl, 20 mM CaCl_2_, and 0.02 % NaN_3_). Gels were stained with 0.1 % R-250 brilliant blue (Boehringer Manheim, IN, USA); 1 μg of conditioned medium from U-937 promyelocyte cells was used in each gel as an indicator of activity.

### Statistical analyses

Descriptive statistics (mean, standard deviation, standard error, median, and range) were obtained for each variable. Data distribution was tested for normality using Kolmogorov-Smirnoff and Shapiro-Wilks tests. When distribution was normal, Student’s t-test was used to analyze for differences among groups. Man-Whitney’s U test was used when data were not normally distributed; a *P* < 0.05 was considered statistically significant.

## Results

With the aim of standardizing our experimental model, we decided to perform a viability assay to demonstrate that the metabolic viability of the placenta explants remained without significant changes along the four days. Taking into account the results obtained from the time-course curve, the LPS-induced cytokines secretion was maximal at 24 h after stimulation (data not shown).

Once concluded the co-stimulations with LPS and P4, we evaluated the secretion patterns of all analytes in the culture medium. Data are presented as mean ± SEM from 10 separate experiments performed in triplicate.

**Fig. 1:**
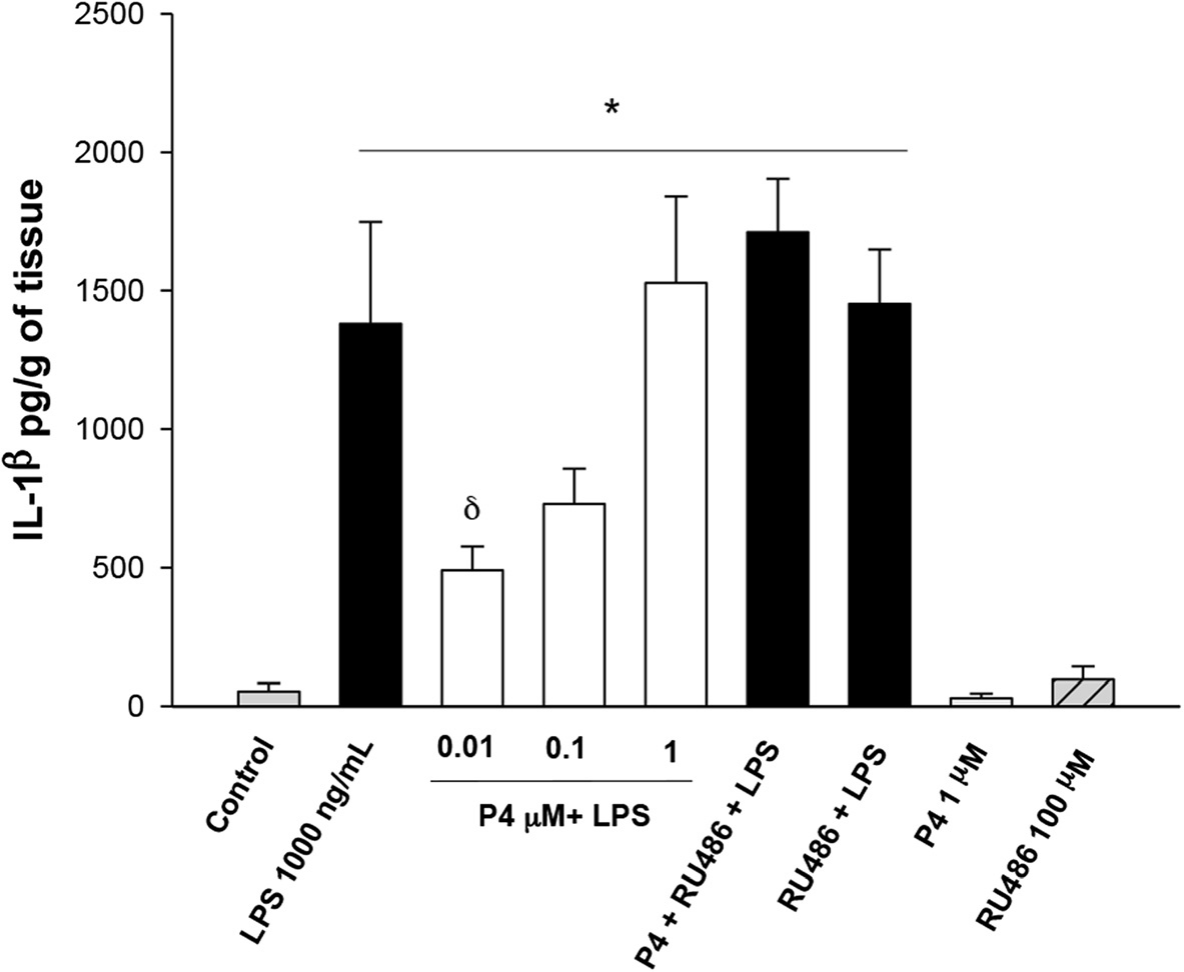
In vitro secretion profile of IL-1β in human placental explants. IL-1β was measured by ELISA in the cultured medium in the basal condition and with different treatments. Data represent 10 independent experiments ± S.E.M., performed in triplicate *P* ≤ 0.05 * versus control; δ versus LPS treatment

Stimulation with LPS enhanced IL-1β secretion 26-times in comparison to basal level (53.0 ± 29.6 pg/g of tissue) and the co-stimulation with 0.1 μmol/L P4 blunted by 56 % this level (606.2 ± 110.9 pg/g of tissue). This effect was reverted by adding the anti-progestin RU486 (1710.85 ± 193.35 pg/g of tissue) (Fig. 1).

**Fig. 2:**
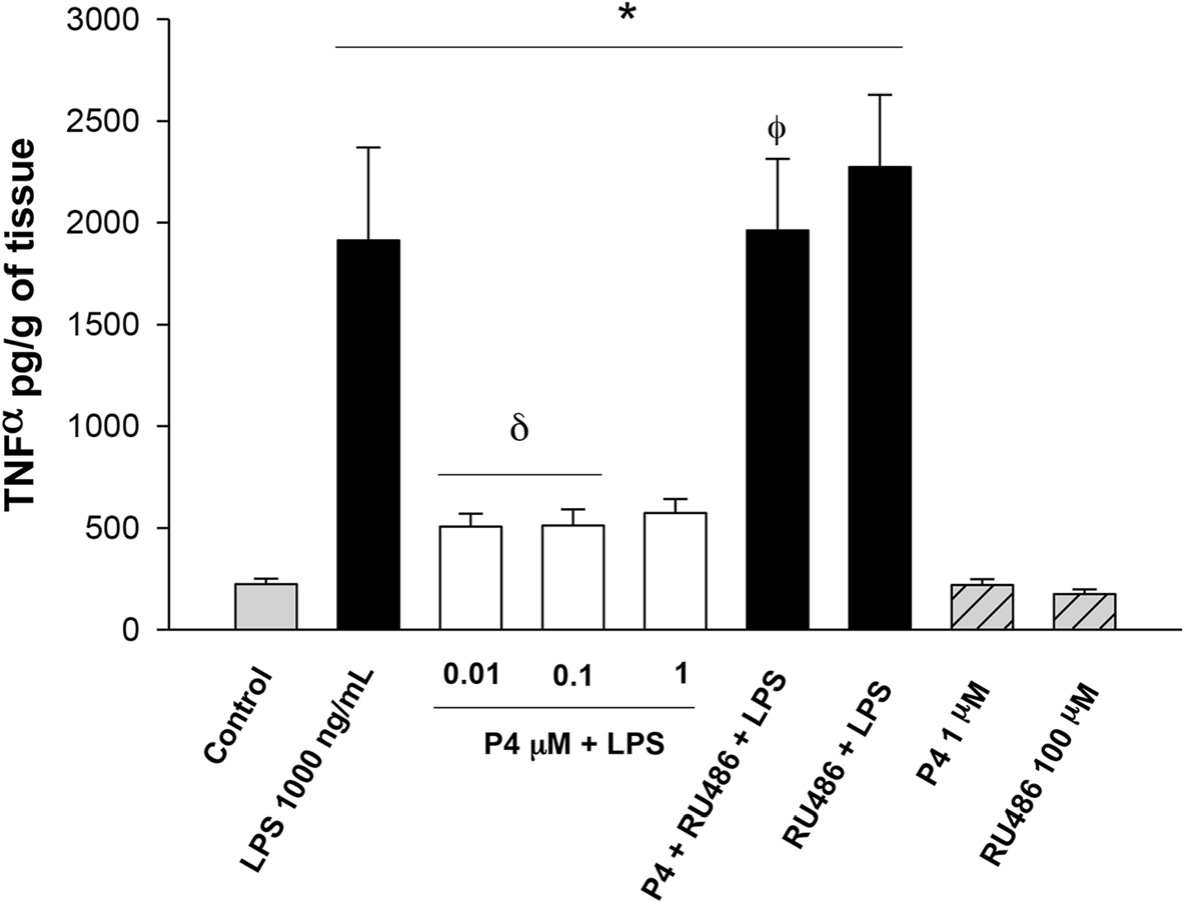
In vitro secretion profile of TNFα in human placental explants. TNFα was measured by ELISA in the cultured medium in the basal condition and with different treatments. Data represent 10 independent experiments ± S.E.M., performed in triplicate *P* ≤ 0.05 * versus control; δ versus LPS treatment; Φ versus 1 μM P4 treatment

In comparison to the basal level of TNFα (224.15 ± 26.2 pg/g of tissue), the culture of placenta explants with 1000 ng/ml LPS induced a significant increase (1912.73 ± 457.25 pg/g of tissue) equivalent to 8-times. The co-addition of P4 (0.01 μM) decreased TNFα by 73 %, an effect that was blocked with 100 μM RU486 (1961.7 ± 351.92 pg/g of tissue) (Fig. 2).

**Fig. 3:**
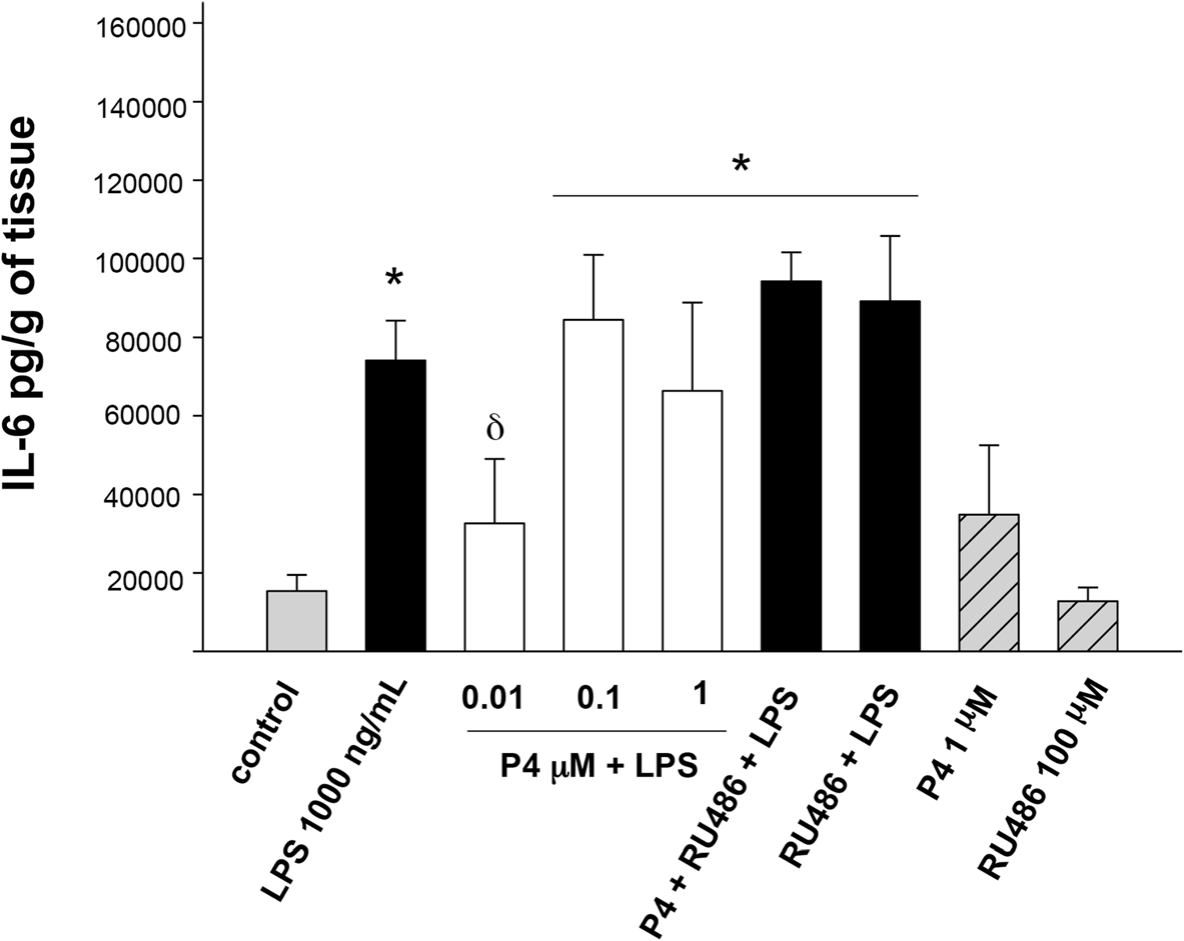
In vitro secretion profile of IL-6 in human placental explants. IL-6 was measured by ELISA in the cultured medium in the basal condition and with different treatments. Data represent 10 independent experiments ± S.E.M., performed in triplicate P ≤0.05 * versus control; δ versus LPS treatment

Basal IL-6 level of the explants was 15,357 ± 4118 pg/g of tissue, stimulation with LPS increased it 4.8-times (74,110 ± 10,154 pg/g of tissue), treatment with P4 inhibited the LPS-induced increase in 55.9 % (32,638 ± 16,336 pg/g of tissue) (Fig. 3).

**Fig. 4:**
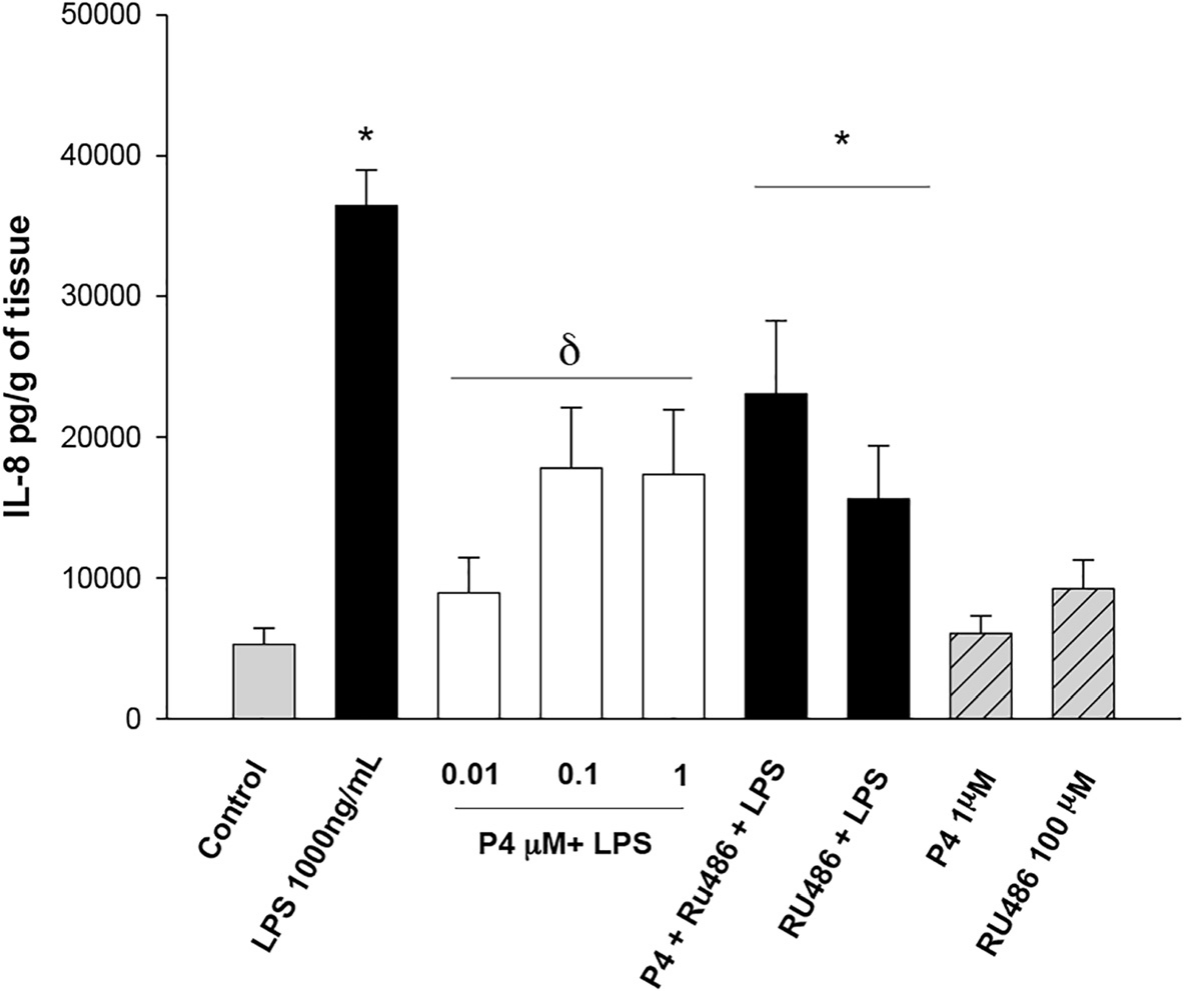
In vitro secretion profile of IL-8 in human placental explants. IL-8 was measured by ELISA in the cultured medium in the basal condition and with different treatments. Data represent 10 independent experiments ± S.E.M., performed in triplicate. *P* ≤ 0.05 * versus control; δ versus LPS treatment

**Fig. 5:**
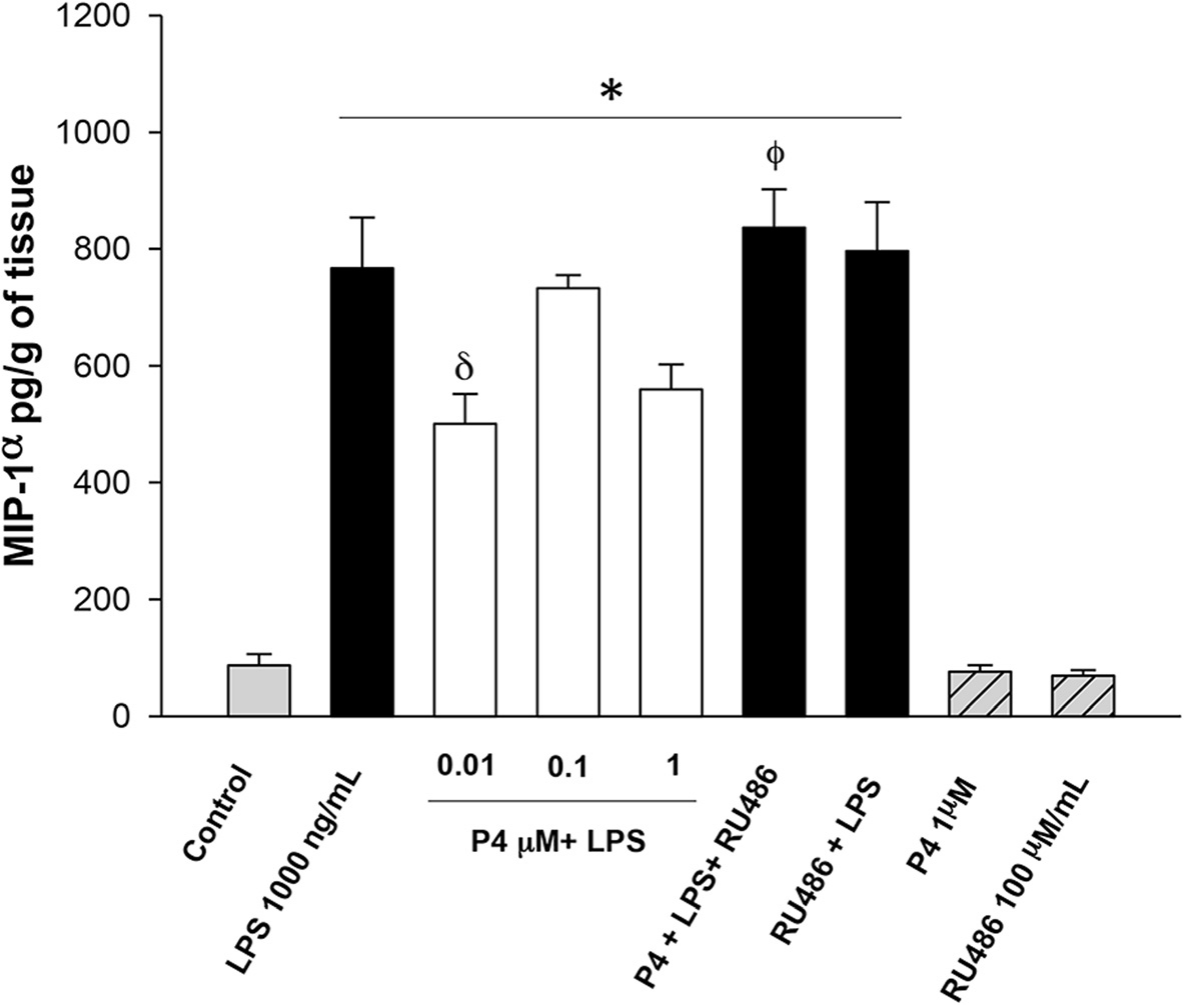
In vitro secretion profile of MIP-1α in human placental explants. MIP-1α was measured by ELISA in the cultured medium in the basal condition and with different treatments. Data represent 10 independent experiments ± S.E.M, performed in triplicate. *P* ≤ 0.05 * versus control; δ versus LPS treatment; Φ versus 1 μM P4 treatment

After stimulation with the bacterial endotoxin, the chemokines IL-8 and MIP1-α increased 7-fold (36,451 ± 2538.6 pg/g of tissue) and 5-fold (766.65 ± 87.34 pg/g of tissue), respectively. Co-stimulation with the three concentrations of P4 inhibited IL-8 (Fig. 4), however MIP-1α was only inhibited by 0.01 μM of P4 (Fig. 5).

**Fig. 6:**
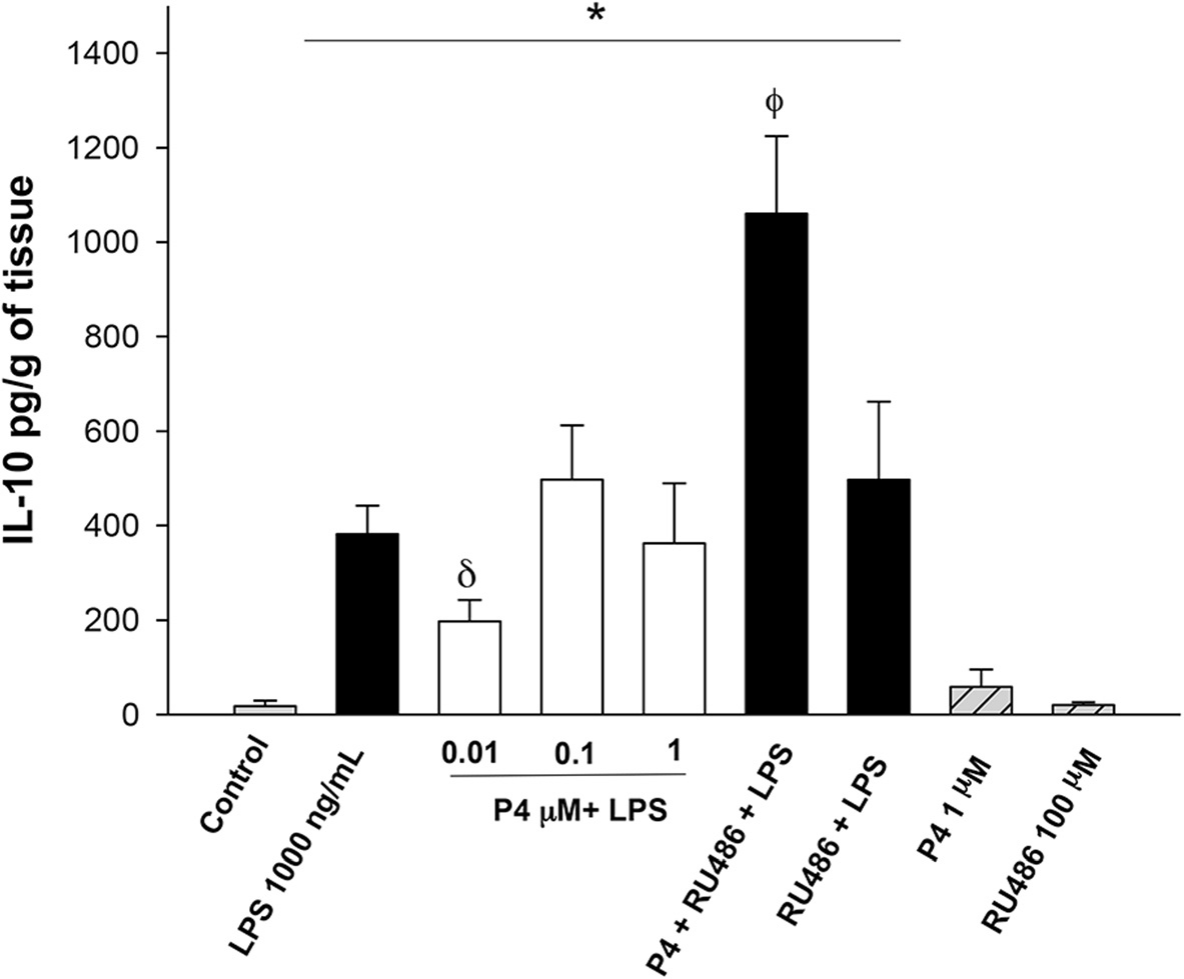
In vitro secretion profile of IL-10 in human placental explants. IL-10 was measured by ELISA in the cultured medium in the basal condition and with different treatments. Data represent 10 independent experiments ± S.E.M., performed in triplicate P ≤ 0.05 * versus control; δ versus LPS treatment; Φ versus 1 μM P4 treatment

The secretion pattern of IL-10 was also modified after stimulation with LPS (381.5 ± 60.21 pg/g of tissue), which represents 21-times the basal level (17.87 ± 11.57 pg/g of tissue). The effect of the endotoxin was inhibited in a significant way when the explants were co-stimulated with 0.01 μM of P4 (197.33 ± 44.49 pg/g of tissue) (Fig. 6).

**Fig. 7:**
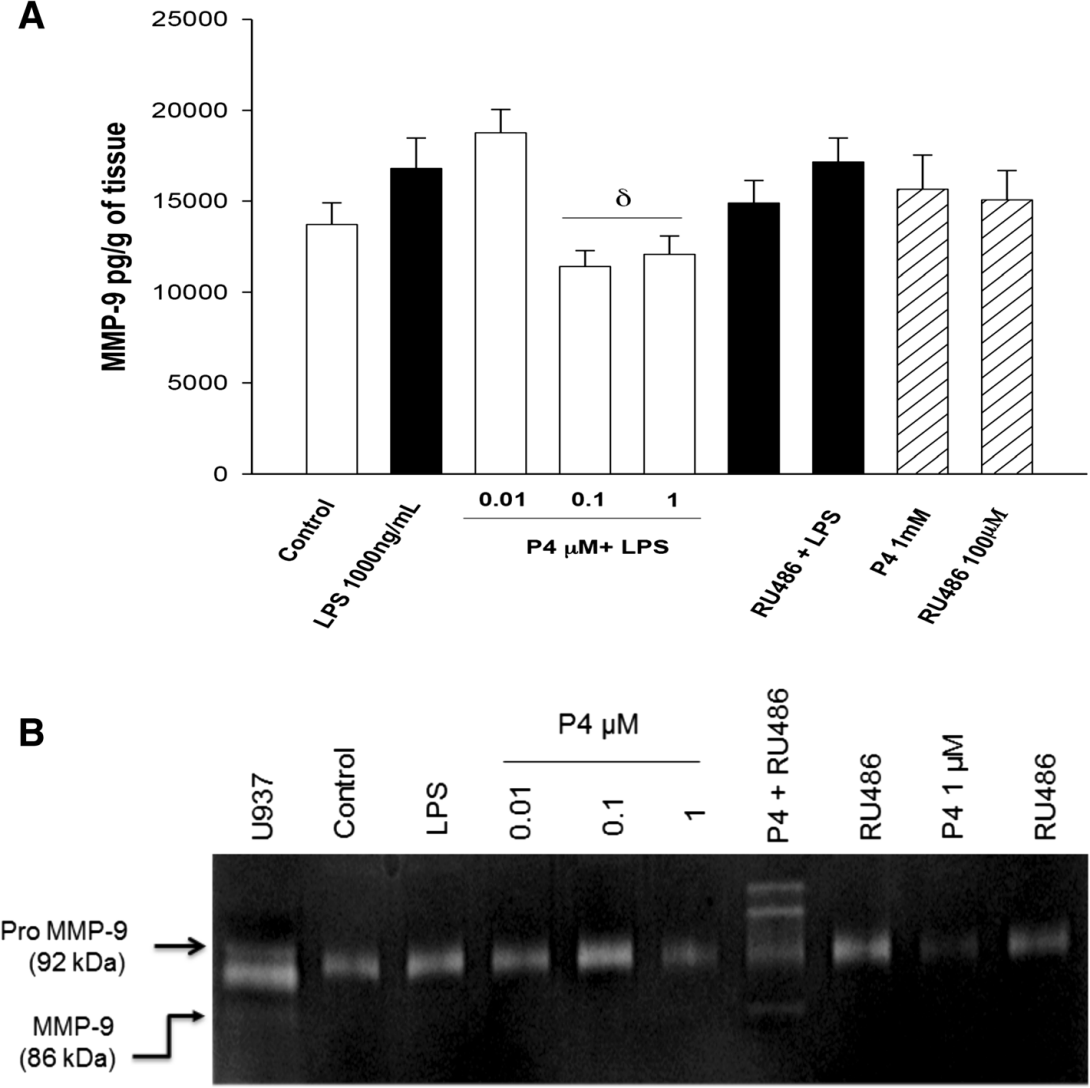
**a** In vitro secretion profile of MMP-9 in human placental explants. MMP-9 was measured by ELISA in the cultured medium in the basal condition and with different treatments. Data represent 10 independent experiments ± S.E.M., performed in triplicate. *P* ≤ 0.05 δ versus LPS treatment. **b** Representative zymogram showing the gelatinase activity present in the cultured medium obtained after each treatment

In comparison to the level of MMP-9 when the same explants were only stimulated with the endotoxin (16,802.6 ± 1672.0 pg/g of tissue), the co-stimulation of explants with 1000 ng/mL of LPS and 0.1 μM of P4 blunted the level of MMP-9 (11,392 ± 976 pg/g of tissue), the equivalent to 1.3 times (Fig. 7a); the gelatinase activity profile shown in the zymogram supports this finding (Fig. 7b).

## Discussion

Successful pregnancy is the result of different immune-endocrine strategies that permit the co-existence of mother and fetus. Based on clinical, experimental and epidemiological evidence, the inflammation associated with an infection process can be considered as one of the most important causes of preterm labor. The mother-fetus co-existence can be compromised if the maternal-fetal milieu is modified by pro-inflammatory modulators that can exert strong effects on the conceptus.

Under exceptional conditions, such as infection, the maternal-fetal unit displays a set of compensatory mechanisms that could eventually limit –partially– damage, and thereby privilege the continuity of pregnancy. One of these mechanisms includes the immunomodulatory effects of P4, considered a key factor in the regulation of the Th1/Th2 balance required to maintain the immune privilege.

On the other hand, an important body of evidence indicates that the placenta is a source of proinflammatory cytokines, such as IL-1β, TNFα, IL-6, which are secreted under basal conditions and in response to different kind of immunologic stimulus [17].

The present results indicate that the stimulation of explants of human villous placenta with LPS increased significantly the level of IL-1β, this cytokine by itself can induce deep changes in the fetal-maternal unit creating conditions incompatible with gestation continuity [18]. Our results are also supported by previous evidence indicating that the chorion of fetal membranes, a region rich in trophoblasts, is the principal source of IL-1β when stimulated selectively with different pathogens associated with preterm labor, including *E. coli* [19], Group B *Streptococcus* [20], and *Gardnerella vaginalis* [21].

In the human term and preterm placenta, IL-1β is secreted basally and after perfusion with LPS of epithelial cells of the amnion, chorion, syncytiotrophoblasts, and stromal cells of villous tissue and the decidua [22]. Furthermore, evidence from animal models support that elevation of IL-1β in the fetal-maternal environment may be an important factor in the pathogenesis of preterm labor associated with intra-amniotic infection [23, 24].

Herein, we demonstrated that pre-stimulation with P4 reduces the secretion of IL-1β induced by the LPS; these results are concurrent with evidence generated in related tissues, such as the amnion epithelium [25], and in a model of choriodecidual infection [26].

Studies done with lymphocytes isolated from women with recurrent miscarriage indicate that dydrogesterone inhibits the production of INF-^γ^, TNFα, IL-4, and IL-6 modifying the Th1/Th2 ratio [27]. Furthermore, at a concentration similar to that found in umbilical cord blood, P4 inhibits cytokine production by cord blood mononuclear cells [28].

TNFα is a key cytokine in the proinflammatory response of the fetal-placental unit under normal and pathological conditions, its concentration has been found elevated in the amniotic fluid of patients with intra-amniotic infection and preterm labor [29].

There is evidence that TNFα is powerful enough to potentiate other inflammatory modulators and to induce preterm labor, fetal injury, and histological chorioamnionitis in a nonhuman primate model [30]. As expected, in our model, TNFα was secreted by explants after stimulation with LPS, which has been previously reported in different experimental models [23, 31].

On the other hand, P4 is able to inhibit the secretion and toxic effects of infection-induced TNF-α in both fetal mononuclear cells isolated from umbilical cord blood and peripheral blood mononuclear cells (PBMCs) from women with unexplained recurrent miscarriage [27], as well as in human monocytes stimulated with heat-killed *Escherichia coli* or *Ureaplasma urealyticum* [32], and in human fetal membranes that are also sensitive to the immunomodulatory effects of P4 [25, 26].

In this work, we also demonstrate that the inhibition of TNF-α by P4 is blocked by RU-486, which suggests that P4 could be acting through both the progesterone receptor (PR) [33–35] and the glucocorticoid receptor (GR) [36, 37], which are present in the human placenta.

Clinical and experimental evidence supports that elevated early second-trimester amniotic fluid IL-6 levels are associated with preterm delivery and can be used as an intrauterine inflammation predictor [38, 39].

Stimulation of explants of placenta with LPS increased IL-6 secretion, and pre-stimulation with P4 impacted the placenta explants inhibiting this effect. The capacity of P4 to limit the secretion of IL-6 has been reported in reproductive tissues such as whole human fetal membranes [26], amnion epithelium [25], myometrium [40], and human uterine cervical fibroblasts [41].

To create a more competitive/effective immune response in the fetal-placental unit undergoing an infectious process, the secretion of chemokines, such as IL-8 and MIP-1α, can attract immune cells that support and enhance the response.

During chorioamnionitis, IL-8 is indispensable in the process of neutrophil infiltration of the decidua [42]. Additional evidence supports that stimulation of human placental multipotent mesenchymal stromal cells with LPS induces the secretion of IL-8, which has been ascribed a potent role in both neutrophil chemotaxis and reduction of neutrophil apoptosis [43].

Using an in vitro culture system in which human umbilical vein endothelial cells constitutively express human PR, Goddard et al. demonstrated that P4 can inhibit the secretion of IL-6, IL-8, CXCL2/3, and CXCL1 induced by LPS [44].

Regarding MIP-1α, there is evidence indicating that this chemokine is produced by trophoblast cells in human placenta [45]; however, it is undetectable in most amniotic fluids from patients in the mid-trimester of pregnancy and at term not in labor [46]. The concentration of MIP-1α correlates with IL-8 and both chemokines increase in the amniotic fluid during microbial invasion of the amniotic cavity [46] and in human fetal membranes during labor [47].

Herein we report the induction of MIP-1α after stimulation with LPS, which has been previously reported in human PBMCs [48, 49]. On the other hand, although there is no information about the effect of P4 on MIP-1α regulation; evidence from other experimental models supports that MIP-1α secreted by CD8+ T lymphocytes is blunted by this steroid hormone [50]. Additionally, it has been reported that the expression of this chemokine can be inhibited also by the treatment of human monocytes and alveolar macrophages with corticosteroids [51].

A key mechanism that modulates the immune equilibrium during pregnancy is IL-10, a cytokine with anti-inflammatory properties that plays pivotal roles in immune recognition and maintenance of gestation, limiting the harmful effects of proinflammatory modulators. IL-10 is produced by immune cells such as T cells, B cells, and macrophages [52–54], as well as by maternal and fetal tissues including the human chorion, the decidua, and the placenta [55–61].

The placenta is an essential tissue for IL-10’s contribution to the maternal-fetal unit, additionally to its role as an immunomodulatory factor, IL-10 is also an important mediator in placental growth and remodeling; changes in its production profile have been associated with labor [62]. The capacity of IL-10 to limit the cytotoxic effects of inflammation is evidenced by the diminution of its concentration associated with labor [63].

The production of IL-10 can be modulated by different stimuli, including proinflammatory cytokines and bacterial products, as well as different pathogens associated with intrauterine infections [64]. Experimental evidence suggests that the choriodecidual region of the fetal membranes is the principal source of this cytokine and stimulation with *E. coli* increases IL-10 [19].

As expected, the present study demonstrates that LPS stimulation induced a significant increase of IL-10, which was dampened by the co-stimulation with P4. The explanation for these results could be controversial. A previous study demonstrated that P4 inhibits the LPS-induced pro-inflammation in a model of choriodecidual infection [26]; however, there is another study in which co-stimulation of fetoplacental artery explants with P4 did not inhibit the LPS-induced IL-10 secretion [65]. These latter results agree with results published by Olmos-Ortiz et al. [66], who demonstrated that IL-10 inhibits placental antimicrobial peptides that, eventually, could modify the entire innate response of the placenta [66].

Stimulation of PBMC from women with recurrent miscarriage with P4 did not modify the secretion profile of IL-10 [27]. These results are not concurrent with clinical studies that demonstrate that dydrogesterone treatment of patients with threatened preterm delivery induces the increase of IL-10 in serum, which is associated with increased length of gestation [67]; in turn, supporting the association between high levels of IL-10 and successful pregnancy.

Once the inflammation modulators are secreted in response to an immunological/infectious stimulus, cytokines such as IL-1β, TNFα, and IL-6 induce the synthesis and secretion of effector modulators, such as MMP-9, which can degrade type IV collagen and gelatin, which are essential in the structure of different tissues of the fetal-placental unit [16, 68].

Many observations suggest that alteration of the equilibrium between the synthesis and degradation of extracellular matrix is a mechanism through which the structural continuity and function are deeply modified during labor under normal and pathological conditions.

In the present study, we demonstrated that the stimulation of placenta explants with LPS induces the increase of MMP-9, this finding is concurrent with evidence obtained from human fetal membranes stimulated with different pathogens [69, 70], supporting that this enzyme is part of the response against different pathogens including *Candida albicans*. Additionally, clinical evidence supports that MMP-9 is an enzyme that increases in the amniotic fluid of women with preterm labor and suspected intra-amniotic infection [71].

Our results show that the pre-stimulation of explants with the highest concentration of P4 inhibits the LPS-induced MMP-9. This could be partially explained by the evidence supporting that different proinflammatory cytokines can induce expression of MMP-9, this cumulative effect impacts the expression of this enzyme by the tissue and a more potent stimulus with P4 than used for inhibition of cytokines is required to induce its inhibition [72].

In this context, previous experimental evidence demonstrated that P4 inhibits other MMPs in term decidual cells, such as MMP-1 and MMP-3 [73] that are key elements during labor, and suppresses the production of pro-MMP-9 induced by IL-1α in rabbit uterine cervical fibroblasts [74].

Experimental and clinical evidence indicates that microbial-induced preterm labor is mediated by an inflammatory process; microorganisms and their products are sensed by pattern recognition receptors, such as Toll-like receptors (TLRs), which induce the production of chemokines (e.g., IL-8 and C-C motif ligand 2 [CCL”]), cytokines (e.g., IL-1β and TNF), prostaglandins, and proteases leading to activation of the common parturition pathway [75–77].

The anti-inflammatory effect of P4 might be exerted through the modulation of immune innate factors, specifically the (TLR)-4, which is constitutively expressed by the human placenta during gestation [78] and is critical for a host inflammatory response to Gram-negative organisms.

Reports from a murine experimental model support that pre-treatment with MPA decreases the LPS-induced up-regulation of TLR-4 mRNA in the cervix and placenta [79]; additionally, stimulation of the human amnion with P4 blunts the inflammation induced by LPS through the inhibition of expression and activation of TLR-4/MyD88 [25]. A similar mechanism could be exerted in the human placenta, however, more studies are required to understand the complexity of signals turned on by this tissue during a scenario complicated by an infectious process.

The effect of P4 described herein supports the concept that the immune-endocrine regulation is key in the maintenance of the immune privilege of the fetal-placental unit. We propose that, under in vivo conditions, P4 can be a mechanism that could limit –partially– the deleterious effects of inflammation. However, its therapeutic use can only be attempted after finding equilibrium between the “protective” anti-inflammatory action and a possible deleterious effect when a strong response against infection is required.

## Conclusion

In summary, results show that P4 reduces the secretion of pro-inflammatory cytokines IL-1β, TNF-α and IL-6, chemokines MIP1α and IL-8, the anti-inflammatory IL-10 as well as the MMP-9. These data suggest that P4 in the placental-fetal unit can be part of an immunomodulatory mechanism that can limit –partially– the deleterious effects of these modulators.

## Abbreviations

LPS: Lipopolysaccharide
P4: Progesterone
IL: Interleukin
MMP: Matrix Metalloprotease
TNF: Tumor necrosis factor
hAbs: Anti-human antibodies
MPA: Medroxyprogesterone acetate
S.E.M: Standard error of the mean
